# Implementing training and support, financial reimbursement, and referral to an internet-based brief advice program to improve the early identification of hazardous and harmful alcohol consumption in primary care (ODHIN): study protocol for a cluster randomized factorial trial

**DOI:** 10.1186/1748-5908-8-11

**Published:** 2013-01-24

**Authors:** Myrna N Keurhorst, Peter Anderson, Fredrik Spak, Preben Bendtsen, Lidia Segura, Joan Colom, Jillian Reynolds, Colin Drummond, Paolo Deluca, Ben van Steenkiste, Artur Mierzecki, Karolina Kłoda, Paul Wallace, Dorothy Newbury-Birch, Eileen Kaner, Toni Gual, Miranda GH Laurant

**Affiliations:** 1 Radboud University Nijmegen Medical Centre, Scientific Institute for Quality of Healthcare (IQ healthcare), P.O. Box 9101, 114 IQ healthcare, 6500 HB Nijmegen, The Netherlands; 2 Institute of Health and Society, Medical Faculty, Baddiley-Clark Building, Richardson Road, Newcastle upon Tyne, NE2 4AX, United Kingdom; 3 Department of Social medicine, University of Gothenburg, P.O. Box 453, 405 30, Gothenburg, Sweden; 4 Department of Medicine and Health, Linköping University, 581 83, Linköping, Sweden; 5 Program on Substance Abuse, Public Health Agency, Government of Catalonia, Barcelona, Spain; 6 Hospital Clínic de Barcelona, Addictions Unit, Institut Clínic de Neurosciències, C/Villarroel 170, CP 08036, Barcelona, Spain; 7 Addictions Department, National Addiction Centre, Institute of Psychiatry, King’s College London, 4 Windsor Walk, London, SE5 8BB, UK; 8 Department of General Practice, Maastricht University, School CAPHRI, P.O Box 616, 6200 MD, Maastricht, The Netherlands; 9 Independent Laboratory of Family Physician Education, Pomeranian Medical University in Szczecin, ul. Rybacka 1, 70-204, Szczecin, Poland; 10 Department of Primary Care and Population Health, University College London, London, UK

**Keywords:** Alcohol, Screening, Brief interventions, Primary healthcare, Training and support, Financial reimbursement, Internet, Implementation

## Abstract

**Background:**

The European level of alcohol consumption, and the subsequent burden of disease, is high compared to the rest of the world. While screening and brief interventions in primary healthcare are cost-effective, in most countries they have hardly been implemented in routine primary healthcare. In this study, we aim to examine the effectiveness and efficiency of three implementation interventions that have been chosen to address key barriers for improvement: training and support to address lack of knowledge and motivation in healthcare providers; financial reimbursement to compensate the time investment; and internet-based counselling to reduce workload for primary care providers.

**Methods/design:**

In a cluster randomized factorial trial, data from Catalan, English, Netherlands, Polish, and Swedish primary healthcare units will be collected on screening and brief advice rates for hazardous and harmful alcohol consumption. The three implementation strategies will be provided separately and in combination in a total of seven intervention groups and compared with a treatment as usual control group. Screening and brief intervention activities will be measured at baseline, during 12 weeks and after six months. Process measures include health professionals’ role security and therapeutic commitment of the participating providers (SAAPPQ questionnaire). A total of 120 primary healthcare units will be included, equally distributed over the five countries. Both intention to treat and per protocol analyses are planned to determine intervention effectiveness, using random coefficient regression modelling.

**Discussion:**

Effective interventions to implement screening and brief interventions for hazardous alcohol use are urgently required. This international multi-centre trial will provide evidence to guide decision makers.

**Trial registration:**

ClinicalTrials.gov. Trial identifier: NCT01501552

## Background

The European Union (EU) has the highest alcohol consumption of the world: in 2009, the average adult (aged 15+ years) alcohol consumption in the EU was 12.5 litres of pure alcohol [1]. A review showed that consumption above 20 to 30 grams of alcohol a day (two to three glasses of wine) increases an individual’s risks of mortality and morbidity [2,3]. However, people often overestimate the positive health effects of alcohol; in fact, only small amounts of alcohol have positive effects on health [2,3].

Alcohol consumption is the third world leading cause of diseases and premature death [1]. The costs related to alcohol are €125bn a year for health, welfare, employment, and criminal justice sectors as a consequence of alcohol-attributable disease, injury, and violence [4]. Therefore, individuals and society would benefit from effective preventive measures with respect to morbidity and mortality and social costs.

There is considerable evidence showing that early identification of hazardous and harmful alcohol consumption result in reduced alcohol consumption and improved health outcomes. Primary healthcare (PHC) is the primary point of contact for many people seeking healthcare. In this setting, screening [5] and brief intervention programs have proven to be effective in reducing alcohol consumption [6-10], with a mean reduction of 38 grams of alcohol per week (three to four glasses of wine) [10]. Although the evidence is still inconsistent about positive effects of nurse-led interventions [11,12], generally screening and brief interventions are provided by healthcare workers such as GPs, nurses, or psychologists [10]. The number needed to treat (NNT) in offering screening and brief interventions is eight (for every eight people treated one will change their behaviour) [13], which is relatively low compared to smoking cessation, which has a NNT of around 35 or higher [14]. Despite the evidence for efficacy and cost-efficacy of screening and brief interventions in PHC, these interventions are rarely implemented in routine practice [15]. Commonly, less than 10% of the population at risk are identified, and less than 5% of those who could benefit are offered screening and brief interventions in PHC settings [15].

Some of the reasons for this gap are identified and can be categorised in three main domains. First, evidence suggests there is substantial lack of knowledge among general practitioners (GPs) [5,16]. A survey across 13 countries found that one-third of all GPs reported never receiving alcohol-related education, 23% reported less than four hours, and 37% reported more than seven hours of alcohol-related education ever [17]. A recent update from England has shown that 52% of the United Kingdom’s surveyed GPs indicated that they had received less than four hours of post-graduate training, continuing medical education, or clinical supervision on alcohol and alcohol related problems [18]. Furthermore a lack of role security and therapeutic commitment has been identified [19].

Secondly, lack of adequate resources and support are identified as important barriers [16,20]. Financial reimbursement could be important measures to overcome this barrier, but as far as we know, there have been no randomised controlled trials conducted investigating the impact of reimbursement for alcohol screening and brief interventions in the PHC setting.

The third important barrier relates to time constrains in terms of perceived workload and work pressure for screening and brief intervention activities [5]. In PHC, trained nurses are increasingly involved in preventive care activities and in the management of chronic ill patients due to the increased workload of GPs. It has shown that they provide safe and effective care [21]. This study focuses on all healthcare professionals working at primary healthcare units (PHCUs).

Although previous implementation studies [22,23] have tried to increase screening and brief interventions in primary healthcare, the gap between scientific knowledge and everyday clinical practice remains [24]. With regard to the first category of barriers of knowledge and attitude, earlier studies found that training and support could make GPs even less secure in their work with drinkers, when the training and support does not address prior GP’s attitude in the training and support [15,19]. In the ODHIN study, we will tailor our implementation strategy to the primary healthcare worker’s prior attitude. With regard to the second category, lack of resources, there are mixed results of evidence of finance systems to change provider behaviour [25,26]. There is limited evidence that finance systems can change provider behaviour of screening and brief interventions of alcohol [15]. Still, financial incentives for smoking cessation interventions have shown a significant positive outcome on increases in referral to tobacco cessation services [27], and suggest financial support for alcohol interventions might be effective. In the third category, workload and work pressure, we suggest e-health interventions might be of benefit. E-self help interventions without therapist support are available both in brief and more extended formats and have shown to be effective in reduction of alcohol consumption [28]. Additionally, internet interventions with therapist support focused on depression and anxiety were found to have larger effect sizes compared to internet interventions without therapist support [29], but has not yet been tested for alcohol internet interventions. These e-health interventions might be helpful to reduce workload of healthcare professionals after identification of patients at risk as well as availability for patients 24 h a day. Therefore, it is of interest to test if primary healthcare workers’ referral to internet-based brief interventions, hereafter termed e-BI, could be time-saving for healthcare professionals and consequently might raise primary care worker’s intervention activity.

It is of significant public health interest to explore, and optimize, effective implementation strategies to improve PHC activities in screening and brief interventions for hazardous and harmful alcohol consumption. In the current study, we evaluate the effect of three strategies, each aimed to tackle one of the above reported barriers, singly or in combination, in order to overcome the gap between knowledge and daily practice.

### Aim and objectives

Our aim is to study the effectiveness of training and support (T&S), financial reimbursement, and internet based brief interventions (e-BI), targeted singly or in combination to primary healthcare units, on screening and brief intervention activities, compared to treatment as usual. The following hypotheses will be tested in the study:

1. Provision of training and support to primary healthcare providers will increase use of preventive screening and brief interventions compared to a care as usual control group.

2. Financial reimbursement to primary healthcare providers as a pay-for-performance of brief alcohol interventions will increase screening and brief intervention rates compared to care as usual.

3. Providing resources, *i.e.*, offering referral possibility to an internet-based method of delivering brief intervention, will increase screening and brief intervention rates compared to care as usual.

4. The combination of training and support, financial reimbursement, and e-BI will be more effective in increasing screening and brief intervention rates compared to single-focused implementation strategies.

## Methods

### Design

Our study is designed as a cluster randomized factorial trial. Data from PHCUs in Catalonia, England, the Netherlands, Poland, and Sweden will be combined to examine the effect of three different implementation strategies singly or in combination on screening and brief advice rates for hazardous and harmful alcohol consumption compared with care as usual (controls). In all countries, the complete trial will be conducted between August 2012 and December 2013.

### Participants

PHCUs with approximately 5,000 to 20,000 registered patients will be the unit of randomization and implementation. In Poland, because practitioners normally operate as single-handed entities working with other practitioners in one building, three practitioners and their staff working in one building will be the unit of randomization. PHCUs who agree to participate in the study are volunteers that will be drawn from administrative or academic registries of PHCU at national or regional levels. PHCUs that have current ongoing alcohol-related projects that have a focus on screening and brief interventions, involve GPs and/or nurses, and include one of the ODHIN implementation strategies, will be excluded.

**Figure 1:**
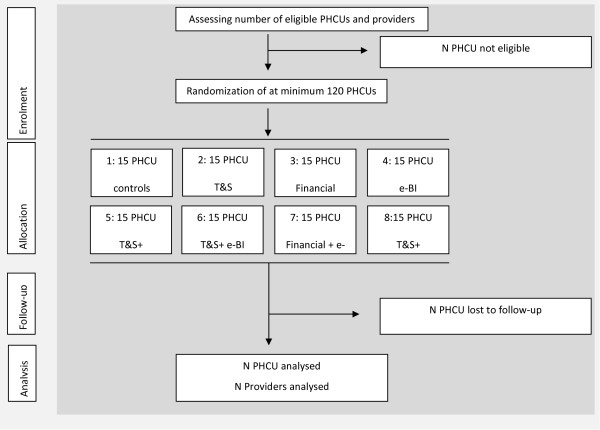
Trial flow chart is required.

Besides fully-trained GPs, nurses or practice assistants with a permanent appointment working in the PHCU and involved in medical and/or preventive care are also eligible, because they also have skills to assist in screening and brief interventions [12,30,31]. At the start of the study, all eligible providers within the PHCU will be identified by the research team. Participating providers are those eligible providers who agree to participate in the trial. Before baseline measurement, the participating providers have to sign up for the study, allowing PHCU with a high number of staff to include only a selection of staff. Staff not able to attend this meeting but willing to participate will be informed by the contact person in the PHCU. These providers will also sign an informed consent for their participation. In the Netherlands, England, Poland and Sweden, PHCU will receive a trial fee. The trial flow chart is shown in Figure 1.

### Implementation strategies

The implementation period will last twelve weeks, with the start date for each country between November 2012 and March 2013. The start date of the implementation period for each PHCU will be staggered. Implementation strategies are outlined in detail in Table 1. All groups will receive the same input as controls but with additional components added. These strategies are about to be tested singly or in combination:

1. Control group, treatment as usual

2. T&S

3. Financial reimbursement

4. e-BI

5. T&S and financial reimbursement

6. T&S and e-BI

7. Financial reimbursement and e-BI

8. T&S, financial reimbursement, and e-BI

**Table 1:** Outline of intervention groups with three different implementation strategies

1.	Control Group—treatment as usual: The control group will receive a package, either hand-delivered or by post, containing a summary card of the national guideline recommendation for screening and brief advice for hazardous and harmful alcohol consumption, without demonstration. In Poland, where no national guidelines exist, the summary card will be adapted from the PHEPA guidelines for the purposes of this trial [33]. No further instructions will be given.
2.	Training and support: Countries differ largely with regard to usual T&S and other educational training of primary care staff. To maximise comparability, a set of minimal and maximal criteria have been established, in which each country specific T&S package should fit.
	In addition to receiving the same package as the control group, the T&S group will be offered two initial 1 to 2 hours face-to-face educational trainings, and one (10 to 30 min) telephone support call to the lead PHCU contact person during the twelve week implementation period. If necessary, one additional face-to-face training of 1 to 2 h duration will be offered. The time intervals between the initial training, the telephone call, and the additional optional training will be, on average, two weeks. The training addresses knowledge, skills, attitudes, and perceived barriers and facilitators in implementing screening and brief advice, combining theory and practical exercises. The location of the educational training will vary from country to country and include in-house meetings at the PHCU or within clusters of PHCUs. The trainers will include peer trainers, members of the research team, accredited teachers, or addiction consultants. Each country will use an adapted existing country-based T&S package. In the case of Poland, the T&S package will be based on the PHEPA training program.
3.	Financial reimbursement: In addition to receiving the same package as the control group, financial reimbursement groups will be paid for their registered screening and brief intervention activities. Payment depends on normal country specific fees and rates for financial reimbursement for clinical preventive activities.
4.	e-BI: In addition to receiving the same package as the control group, the e-BI group will be asked to refer identified at risk patients with an e-leaflet with unique log in codes to an approved e-BI specific package, which will be country specific, or, for Poland based on the WHO e-SBI program. The website should include the following: Log-in facility to allow monitoring of the patient (*i.e.*, patient actually log-in); suitable brief screening tool with ability to calculate score and give feedback (*i.e.*, brief intervention); appropriate information on sensible drinking guidelines; information on impact of alcohol on health and wellbeing; and a drink diary facility. Furthermore, the website could offer reminder facilities for follow-up activity.
5.	T&S and financial reimbursement: The T&S and financial reimbursement group will receive the package, T&S, and the financial reimbursement as described above.
6.	T&S and e-BI: The T&S and e-BI group will receive the package, T&S as above, and will be asked to refer identified at risk patients to e-BI as above.
7.	Financial reimbursement and e-BI: The financial reimbursement and e-BI group will receive the package and will be asked to refer identified at risk patients to e-BI as above. They will be paid for screening, referral performance to e-BI, and brief advice if actually delivered, with the system of pay as above.
8.	T&S, financial reimbursement and e-BI: The T&S, financial reimbursement and e-BI group will receive the package and T&S as above. They will be asked to refer identified at risk patients to e-BI as above. They will be paid for screening, brief advice activities, and referral performance to e-BI, with the system of pay as above.

A graphical depiction of the study is depicted in Additional file 1.

An introductory meeting (first briefing) will be held in all PHCUs that agree to participate in the study, describing the study’s purpose and the four-week baseline data collection, which will follow the introductory meeting. After the baseline data collection, all PHCUs will receive a second briefing within one month, either face to face or by telephone, tailor-made to the study group to which they are allocated.

### Outcomes

#### Primary outcomes: screening and brief advice rates

PHCUs will be asked to screen all patients aged 18 years and over who attended the PHCU. These patients are defined as eligible patients.

Patients will be screened for hazardous and harmful alcohol consumption with AUDIT-C [32]. Screen positives, or at risk patients, are defined as those who scored ≥ 5 for men or ≥ 4 for women on AUDIT-C. Participating staff that have signed up to the study will be asked to deliver brief alcohol advice of 5 to 15 min duration to at-risk patients, with the length and format of the brief advice based on country-specific guidelines or, for Poland where national guidelines are lacking, the European guidelines developed by PHEPA [33]. Providers of PHCUs allocated to e-BI activity will be asked to refer patients to a computerized brief advice program, considered equivalent to providing brief advice. Besides counting referral rate to e-BI, actual e-BI log-in rates of patients will be collected.

Screening and brief advice will be measured at five timepoints: during the four-week baseline period, the three consecutive four-week blocks during the twelve week implementation period; and, the four-week follow-up period, which will occur during the seventh month after the end of the twelve-week implementation period using paper tally sheets, with the exception of Catalonia who will use their electronic patient records. The tally sheets include AUDIT-C scores (*i.e.*, identification of at risk patients) with additional table to indicate the type of brief advice that was delivered to the patients at risk. Gender and age of patients will be recorded as well as the name and profession of the provider.

The screening rate will be calculated as the number of completed screens divided by the total number of consultations of all patients eligible for screening (as defined above) per participating provider times 100. The brief advice rate will be calculated as the number of BIs delivered (received oral brief advice, and/or were given an advice leaflet, and/or were referred to the e-BI program, and/or were referred to another provider in or outside the practice), divided by the total number of screen positives per participating provider times 100. Information will also be collected on the number of screen negatives who received brief advice.

Screening and brief advice rates will be calculated at two levels: at an aggregate PHCU level for all participating providers in the PHCU; and, at an individual provider level for each participating and actively participating providers. Participating providers are defined as those who attended the first briefing, or who were identified as joining the study by the contact person of the PHCU at the first briefing. Actively participating providers are defined as those participating providers who completed at least one tally sheet or computerized record during one of the measurement periods.

#### Secondary outcomes: role security and therapeutic commitment

Role security and therapeutic commitment of the participating providers will be measured by the short version of the Alcohol and Alcohol Problems Perception questionnaire (SAAPPQ) [34] at three time points: at or immediately after the first introduction meeting, at the end of the 12-week implementation period, and during the end of the four-week follow-up period. All participating providers who have signed an informed consent will be asked to complete the SAAPPQ at each of the three time points. The responses will be summed within the two scales of role security and therapeutic commitment. Individual missing values for any of the items in a domain will be assigned the mean value of the remaining items of the domain before summation.

### Randomization and blinding

Randomization will take place after formal agreement of the PHCU to take part in the trial. The PHCU will be randomly allocated to one of eight groups by the European coordinating centre, using computerized randomization stratifying by country, ensuring 15 PHCUs per group (three per country). Although the PHCUs will be randomly allocated before the baseline measurement, the research team in each of the countries and the PHCU only are informed of the allocation after collection of the baseline measurement to avoid bias as a result of group allocation. For the remainder of the study period, the PHCU and investigators will not be blind to group allocation.

### Sample size

It is estimated that 56 PHCUs (seven per eight allocation groups) with a minimum of 1,000 eligible patients per month would be needed for a 80% chance of detecting an increase in screening rates from 8% to 12% (ICC = 0.029) and that 120 PHCUs (15 per eight allocation groups) would be needed for a 80% chance of detecting an increase in brief advice rates from 4% to 6% (ICC = 0.029) (alpha = 5%). As country is used as stratification criteria each country has to include a minimum of 24 PHCUs. These conservative estimates are based on published evidence of screening and advice rates [22,23].

### Statistical methods

Because of the hierarchical structure of the data (individual providers nested within PHCU nested within country), we will perform multilevel analyses of the screening and advice rates to examine the effect of the implementation strategies in comparison with the controls. The intention to treat analyses will include all participating providers (see above). Per protocol analyses will include only actively participating providers (see above). In all the analyses, we will use exposure to the implementation strategy as co-variate. Exposure is defined as positive if the providers meet the following criteria: financial—the PHCU received the financial reimbursement; e-BI—the provider handed out at least one referral card; and T&S—the provider attended the two face-to-face educational meetings. If these criteria will not be met, the exposure will be defined as negative.

Analyses will be performed in SAS V9.2 and based on mixed effects model (PROC GLIMMIX and PROC MIXED). We will use a random intercept model with fixed variables.

## Discussion

By conducting this trial, we are trying to address the well-known implementation gap (evidence to practice) of screening and brief interventions for hazardous and harmful alcohol consumption in PHC. For example, researchers rarely have been in a position to actively compare a number of incentive-based strategies. With this trial, we aim to assist in building a knowledge base, on which policy could be based on.

We are aware of some strengths and limitations of this trial. This trial is approached pragmatically. In other words, each of the five countries differs slightly in the implementation strategy contents. For example, countries will differ in their distribution of research fees, amount of financial reimbursements, and deliverers of training and support strategies. The research team explicitly determined this pragmatic approach, because they considered this approach being most valuable for country policy makers. Albeit, in terms of research, this is less powerful because there are small variations in implementation strategies per country.

The five participating countries are different in their organization of primary care and have different drinking patterns. This creates opportunities to conduct across-country analyses and relate different implementation rate outcomes to cultural and organizational differences. These results can consequently be applicable through Europe and other similar Western countries. In the future, if our implementation strategies result in improved screening and brief intervention rates, other countries with comparable primary care systems could use these strategies to improve the prevention of hazardous and harmful alcohol consumption in their country.

## Abbreviations

e-BI: Internet based brief interventions; PHC: Primary healthcare; PHCU: Primary healthcare unit; SAAPPQ: Short Alcohol and Alcohol Perceptions Questionnaire; T&S: Training and support.

## Competing interests

The authors declare that they have no competing interests.

## Authors’ contributions

MK, PA, and ML wrote the first draft of the manuscript and all other authors revised the manuscript critically. PB, FS, and PA were responsible for the original set up of the study design, which has further been discussed with all authors during three meetings. The final and in this paper presented design is the outcome of these discussions and written comments of all authors. All authors read and approved the final manuscript.

## Supplementary Material

Additional file 1 **Graphical depiction of ODHIN study.** This image graphically describes the way ODHIN is designed. It makes a distinction between procedure activities, measures and implementation strategies. It also gives an insight of time schedule of depicted activities. Click here for file

## References

[B1] WHO Regional Office for Europe Anderson P, Møller L, Galea G Alcohol in the European Union. Consumption, harm and policy approaches 2012 WHO Regional Office for Europe, Copenhagen

[B2] Anderson P Cremona A Paton A Turner C Wallace P The risk of alcohol Addiction 1993 88 1493 1508 10.1111/j.1360-0443.1993.tb03135.x 8286995

[B3] Anderson P Alcohol and risks of physical harm 1995 Oxford Medical Publications, Oxford

[B4] Anderson P Baumberg B Alcohol in Europe 2006 Institute of Alcohol Studies, London

[B5] University of Sheffield Prevention and early identification of alcohol use disorders in adults and young people. Final draft of Report 2 to the National Institute for Health & Clinical Excellence 2009 University of Sheffield: School of Health and Related Research (ScHARR), Sheffield

[B6] Fleming MF Mundt MP French MT Manwell LB Stauffacher EA KL B Brief physician advice for problem drinkers: long-term efficacy and benefit-cost analysis Alcohol Clin Exp Res 2002 26 36 43 10.1111/j.1530-0277.2002.tb02429.x 11821652

[B7] Anderson P Lawrence M, Neil A, Fowler G, Mant D The effectiveness of general practitioners’ advice in reducing the risk of alcohol Prevention of Cardiovascular Disease: An evidence-based approach 1996 Oxford University Press, Oxford

[B8] Babor T Higgins-Biddle J Alcohol screening and brief intervention: dissemination strategies for medical practice and public health Addiction 2000 95 677 686 10.1046/j.1360-0443.2000.9556773.x 10885042

[B9] Fleming MF Mundt MP French MT Manwell LB Stauffacher EA KL B Benefit-cost analysis of brief physician advice with problem drinkers in primary care settings Med Care 2000 38 7 18 10.1097/00005650-200001000-00003 10630716

[B10] Kaner EF Beyer F Dickinson HO Pienaar E Campbell F Schlesinger C Heather N Saunders J Burnand B Effectiveness of brief alcohol interventions in primary care populations Cochrane Database Syst Rev 2007 18 CD004148 17443541 10.1002/14651858.CD004148.pub3

[B11] Lock CA Kaner E Heather N Doughty J Crawshaw A McNamee P Purdy S Pearson P Effectiveness of nurse-led brief alcohol intervention: a cluster randomized controlled trial J Adv Nurs 2006 54 426 439 10.1111/j.1365-2648.2006.03836.x 16671972

[B12] Reiff-Hekking S Ockene JK Hurley TG Reed GW Brief physician and nurse practitioner-delivered counseling for high-risk drinking. Results at 12-month follow-up J Gen Intern Med 2005 20 7 13 10.1111/j.1525-1497.2005.21240.x 15693921 PMC1490034

[B13] Moyer A Finney J Swearingen C Vergun P Brief interventions for alcohol problems: a meta-analytic review of controlled investigations in treatment-seeking and non-treatment-seeking populations Addiction 2002 97 279 292 10.1046/j.1360-0443.2002.00018.x 11964101

[B14] Stead LF Bergson G Lancaster T Physician advice for smoking cessation Cochrane Database Syst Rev 2008 2 CD000165 18425860 10.1002/14651858.CD000165.pub3

[B15] Anderson P Overview of interventions to enhance primary-care provider management of patients with substance-use disorders Drug Alcohol Rev 2009 28 567 574 10.1111/j.1465-3362.2009.00113.x 19737215

[B16] Nilsen P Brief alcohol intervention–where to from here? Challenges remain for research and practice Addiction 2010 105 954 959 10.1111/j.1360-0443.2009.02779.x 20121717

[B17] Kaner EF Wutzke S Saunders JB Powell A Morawski J JC; B, Group WBIS Impact of alcohol education and training on general practitioners’ diagnostic and management skills: findings from a World Health Organization collaborative study J Stud Alcohol 2001 62 621 627 11702801 10.15288/jsa.2001.62.621

[B18] Wilson G Lock C Heather N Cassidy P Christie M Kaner E Intervention against excessive alcohol consumption in primary health care: a survey of GPs’ attitudes and practices in England ten years on Alcohol Alcohol 2011 46 570 577 10.1093/alcalc/agr067 21690169 PMC3156887

[B19] Anderson P Kaner E Wutzke S Funk M Heather N Wensing M Grol R Gual A Pas L Attitudes and managing alcohol problems in general practice: an interaction analysis based on findings from a WHO Collaborative Study Alcohol Alcohol 2004 39 351 356 10.1093/alcalc/agh072 15208170

[B20] Roche AM Freeman T Brief interventions: good in theory but weak in practice Drug Alcohol Rev 2004 23 11 18 10.1080/09595230410001645510 14965883

[B21] Laurant M Reeves D Hermens R Braspenning J Grol R Sibbald B Substitution of doctors by nurses in primary care Cochrane Database Syst Rev 2005 2 CD001271 15846614 10.1002/14651858.CD001271.pub2

[B22] Funk M Wutzke S Kaner E Anderson P Pas L McCormick R Gual A Barfod S A multi country controlled trial of strategies to promote dissemination and implementation of brief alcohol intervention in primary health care: Findings of a WHO Collaborative Study J Stud Alcohol 2005 66 379 16047527 10.15288/jsa.2005.66.379

[B23] van Beurden I Anderson P Akkermans RP Grol RP Wensing M Laurant MG Involvement of general practitioners in managing alcohol problems: a randomised controlled trial of a tailored improvement programme Addiction 2012 107 1601 1611 10.1111/j.1360-0443.2012.03868.x 22372573

[B24] Nilsen P Aalto M Bendtsen P Seppa K Effectiveness of strategies to implement brief alcohol intervention in primary healthcare. A systematic review Scand J Prim Health Care 2006 24 5 15 10.1080/02813430500475282 16464809

[B25] Scott A Sivey P Ait Ouakrim D Willenberg L Naccarella L Furler J Young D The effect of financial incentives on the quality of health care provided by primary care physicians Cochrane Database Syst Rev 2011 9 CD008451 21901722 10.1002/14651858.CD008451.pub2

[B26] van CE D Changing the GP payment system: Do financial incentives matter? PhD thesis 2012 Tilburg University, Nivel

[B27] An LC Bluhm JH Foldes SS Alesci NL Klatt CM Center BA Nersesian WS Larson ME Ahluwalia JS MW M A randomized trial of a pay-for-performance program targeting clinician referral to a state tobacco quitline Arch Intern Med 2008 168 1993 1999 10.1001/archinte.168.18.1993 18852400

[B28] Riper H Spek V Boon B Conijn B Kramer J Martin-Abello K F S Effectiveness of E-Self-help interventions for curbing adult problem drinking: a meta-analysis J Med Internet Res 2011 13 e42 10.2196/jmir.1691 21719411 PMC3221381

[B29] Spek V Cuijpers P Nyklicek I Riper H Keyzer J Pop V Internet-based cognitive behaviour therapy for symptoms of depression and anxiety: a meta-analysis Psychol Med 2007 37 319 328 10.1017/S0033291706008944 17112400

[B30] Kaner E Lock C Heather N McNamee P Bond S Promoting brief alcohol intervention by nurses in primary care: a cluster randomised controlled trial Patient Educ Couns 2003 51 277 284 10.1016/S0738-3991(02)00242-2 14630384

[B31] Israel Y Hollander O Sanchez-Craig M Booker S Miller V Gingrich R Rankin JG Screening for problem drinking and counseling by the primary care physician-nurse team Alcohol Clin Exp Res 1996 20 1443 1450 10.1111/j.1530-0277.1996.tb01147.x 8947323

[B32] Bradley KA DeBenedetti AF Volk RJ Williams EC Frank D Kivlahan DR AUDIT-C as a brief screen for alcohol misuse in primary care Alcohol Clin Exp Res 2007 31 1208 1217 10.1111/j.1530-0277.2007.00403.x 17451397

[B33] Anderson P Gual A Colom J Alcohol and Primary Health Care: Clinical Guidelines on Identification and Brief Interventions 2005 Department of Health of the Government of Catalonia, Barcelona

[B34] Anderson P Clement S The AAPPQ Revisited: the measurement of general practitioner’s attitudes to alcohol problems Addiction 1987 82 753 759 10.1111/j.1360-0443.1987.tb01542.x 3478065

