# Supplementary material for: Implementing training and support, financial reimbursement, and referral to an internet-based brief advice program to improve the early identification of hazardous and harmful alcohol consumption in primary care (ODHIN): study protocol for a cluster randomized factorial trial
Source: Implement Sci. 2013 Jan 24;8:11. doi: 10.1186/1748-5908-8-11 (PMC3564747; doi:10.1186/1748-5908-8-11)
Supplement: Additional file 1 — Graphical depiction of ODHIN study. This image graphically describes the way ODHIN is designed. It makes a distinction between procedure activities, measures and implementation strategies. It also gives an insight of time schedule of depicted activities. [file 1748-5908-8-11-S1.docx]

**Additional file 1: Graphical depiction of the ODHIN study**

| **Time schedule** | ***Period*** | ***Control group*** | ***T&S*** | ***Financial*** | ***e-BI*** | ***T&S+***  ***Financial*** | ***T&S+e-BI*** | ***Financial***  ***+e-BI*** | ***T&S+***  ***Financial***  ***+e-BI*** |
| --- | --- | --- | --- | --- | --- | --- | --- | --- | --- |
| *Baseline:*  1 month | *September 2012 – February 2013* |  |  |  |  |  |  |  |  |
|  |  | a  b | a  b | a  b | a  b | a  b | a  b | a  b | a  b |
| *Randomization* |  | | | | | | | | |
| 1 month | *October 2012 – March 2013* |  |  |  |  |  |  |  |  |
| *Implementation period:*  3 months | *November 2012 – June 2013* | a  b | a  b | a  b | a  b | a  b | a  b | a  b | a  b |
| *Follow-up:*  6 months after implementation finished | *July 2012– December 2013* | a  b | a  b | a  b | a  b | a  b | a  b | a  b | a  b |

*Legend:*

*T&S = Training and support; Financial = financial reimbursement; e-BI = internet-based brief intervention*

*Procedure activities*

|  | *1) 30 min introduction to study*  *2) 15-45 min meeting introduction to study arms (within control and financial reimbursement only arm- briefing either face to face or by telephone)* |
| --- | --- |

*Measures*

|  | *a) Collecting tally sheets of SBI activity (baseline and follow-up 1 month; implementation period 3 months)*  *b) SAAPPQ measurement* |
| --- | --- |
